# Coagulansin-A has beneficial effects on the development of bovine embryos *in vitro* via HSP70 induction

**DOI:** 10.1042/BSR20150222

**Published:** 2016-03-16

**Authors:** Imran Khan, Kyeong-Lim Lee, Md. Fakruzzaman, Seok-Hwan Song, Ihsan-ul-Haq, Bushra Mirza, Chang Guo Yan, Il-Keun Kong

**Affiliations:** * Department of Animal Science, Division of Applied Life Science (BK21 Plus), Gyeongsang National University, Jinju 660-701, Gyeongnam Province, Republic of Korea; † Department of Pharmacy, Faculty of Biological Sciences, Quaid-i-Azam University, Islamabad 45320, Pakistan; ‡ Department of Biochemistry, Quaid-i-Azam University, Islamabad 45320, Pakistan; § Jilin Co-Innovation Center of Beef Cattle Science and Industry Technology, Yanbian University, Yanji, Jilin 133002, P.R. China; ║ Institute of Agriculture and Life Science, Gyeongsang National University, Jinju 660-701, Gyeongnam Province, Republic of Korea

**Keywords:** bovine embryo, coagulansin-A, DNA damage, heat shock protein 70 (HSP70), nuclear factor-κB (NF-κB)

## Abstract

Coagulansin-A (withanolide) is the steroidal lactone obtained from *Withania coagulans* which belong to Solanaceae family. The present study investigated the effects of coagulansin-A on bovine oocyte maturation and embryo development *in vitro*. All these oocytes were aspirated from the ovaries obtained from Korean Hanwoo cows at a local abattoir. To determine whether coagulansin-A has beneficial effects on bovine oocyte maturation *in vitro*, 355 oocytes per group (control and treated) in seven replicates were subjected with different concentrations (1, 2.5, 5, 7.5 and 10 μM) of coagulansin-A. The coagulansin-A was added in the *in vitro* maturation (IVM) media followed by *in vitro* fertilization (IVF) and then *in vitro* culture (IVC). Only treatment with 5 μM coagulansin-A remarkably (*P*<0.05) improved embryos development (Day 8 blastocyst) having 27.30 and 40.01% for control and coagulansin-A treated groups respectively. Treatment with 5 μM coagulansin-A significantly induced activation of heat shock protein 70 (HSP70) (*P*<0.05). Immunofluorescence analysis revealed that 5 μM coagulansin-A treatment also significantly inhibited oxidative stress and inflammation during bovine embryo development *in vitro* by decreasing 8-oxoguanosine (8-OxoG) (*P*<0.05) and nuclear factor-κB (NF-κB) (*P*<0.05)*.* The expressions of HSP70 and NF-κB were also conformed through real-time PCR (RT-PCR). Additionally, the terminal deoxynucleotidyl transferase dUTP nick-end labelling (TUNEL) assay confirmed that coagulansin-A treatment significantly improved the embryo quality and reduced bovine embryo DNA damage (*P*<0.05). The present study provides new information regarding the mechanisms by which coagulansin-A promotes bovine embryo development *in vitro*.

## Abstract (precis)

Treatment with the steroidal lactone, coagulansin-A, improves bovine oocyte maturation and embryo development *in vitro* by inducing heat shock protein 70 (HSP70), which reduces the levels of reactive oxygen species (ROS), DNA damage and inflammation.

## INTRODUCTION

In assisted reproductive technology (ART), *in vitro* maturation (IVM) is an extremely important step, followed by fertilization and embryo culture. For mammals, culture is normally performed in 5% CO_2_ and 95% air (20% O_2_) [1]. The main difference between *in vivo* and *in vitro* environments is the O_2_ concentration, which is greater in the latter than in the former [2]. The increased O_2_ concentration leads to the production of large amounts of reactive oxygen species (ROS), which have detrimental effects on developing embryos [3]. These effects include DNA and protein damage and activation of various signalling pathways (P53 and P38), leading to apoptosis [4].

Withanolide are steroidal lactones obtained from *Withania* plants, which belong to the Solanaceae family. There are two important *Withania* species, namely, *Withania coagulan* and *Withania somnifera*, which are native to Pakistan and India [5]. Withanolides containing a 14, 20-epoxide bridge are specific to *W. coagulans*. Hepatoprotective, antihyperglycaemic, anti-inflammatory, hypolipidaemic, free radical scavenging, cardiovascular, antimicrobial, central nervous system depressant, cytotoxic, antitumour and immunomodulating activities have been studied in *W. coagulans* [6]. The variety of activities reported for the extracts, fractions and withanolides isolated from *W. coagulans* provide promising evidence for future research. Withanolides are also reported to have vital role against cancer and enhance the apoptosis in cancer cells as well as prevent tumorigenesis by inhibiting tumour necrosis factor-α (TNF-α) activated nuclear factor-κB (NF-κB) [5,7]. Clinical and animal research confirmed that withanolide has positive effects in the treatment of Alzheimer's disease, Parkinson's disease and dementia. It is also used as an anti-ulcer agent, a tonic for liver and an antioxidant due to its scavenging of ROS [8–10]. Derivatives of withanolide have antioxidant properties and reduce lipid peroxidation [11]. Withanolide also inhibits the activities of cyclooxygenase (COX)-1 and -2, which are involved in inflammation [12].

In the present study, we used coagulansin-A isolated from the naturally growing *W. coagulans*, in ART for the first time to test its beneficial effects on bovine embryo development *in vitro*. For this purpose, different concentrations of coagulansin-A (1, 2.5, 5, 7.5 and 10 μM) were tested. Treatment with 5 μM coagulansin-A has more positive effects on embryo development than other concentrations and so we focused on this concentration in comparison with the control group.

## MATERIALS AND METHODS

### Reagents

All chemicals and reagents were obtained from Sigma–Aldrich, unless otherwise noted. Experiments were conducted in accordance with the Gyeongsang National University guidelines for the care and use of laboratory animals (approval no. GAR-110502-X0017).

### Isolation and characterization of coagulansin-A

**Figure 1:**
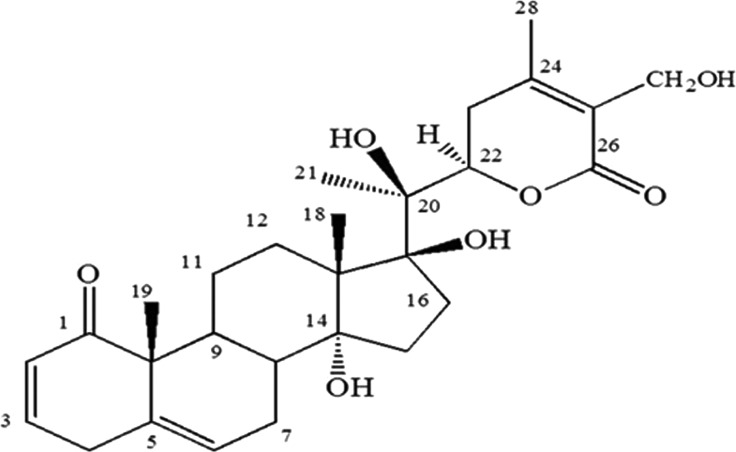
Structure of coagulansin-A [5].

Plant material was collected, sorted out for any foreign material, diseased or deteriorated parts. Then it was shade dried with continuous agitation every 6 hourly and then crushed in a grinding mill. Total 10 kg of shade dried and crushed aerial parts without fruits (leaves and stems only) were taken and macerated in 30 litres of solvent for 3 days by occasional shaking. Mixture of chloroform and methanol (1:1) was used as extraction solvent. Filtrate of extraction was dried by vacuum distillation. It was then subjected to solvent extraction and normal phase preparative chromatography to isolate 50 mg of coagulansin-A. The compound was characterized by performing 2D NMR and LC–MS experiments (Figure 1) [5].

### Experimental design

The ROS-scavenging compound coagulansin-A was added to IVM media in order to overcome the effects of ROS. Embryos were divided into various groups that have 1, 2.5, 5, 7.5 and 10 μM coagulansin-A concentration in treatment 1, treatment 2, treatment 3, treatment 4, treatment 5 and control group (without treating compound) respectively. From these different concentration 5 μM coagulansin-A was selected as an optimal concentration for further study. Real-time PCR (RT-PCR), immunofluorescence technique and TUNNEL were used for developmental analysis in control and coagulansin-A treated groups. The effects of coagulansin-A were determined at a normal culture temperature (38.5°C).

### Cumulus–oocyte complex recovery

Ovaries of Korean native cows (Hanwoo) were collected at a local abattoir, placed in physiological saline (0.9% sodium chloride) at approximately 35°C and transported to the laboratory within 2 h after slaughter. Ovaries were washed in fresh Dulbecco's PBS (D-PBS) and cumulus–oocyte complexes (COCs) were recovered as described by Deb et al. [13]. In brief, COCs were recovered from follicles with a diameter of 2–8 mm using an 18-gauge needle attached to a vacuum pump. Aspirated fluid was expelled into dishes containing TL-HEPES medium (114 mM sodium chloride, 3.2 mM potassium chloride, 2 mM sodium bicarbonate, 0.34 mM sodium biphosphate, 10 mM sodium lactate, 0.5 mM magnesium chloride, 2.0 mM calcium chloride, 10 mM HEPES, 1 μl*/*ml phenol red, 100 IU/ml penicillin and 0.1 mg/ml streptomycin) and imaged with a stereomicroscope. Good-quality oocytes with more than three layers of compact cumulus cells and homogeneous cytoplasm were selected. COCs were washed three times in TL-HEPES medium.

### *In vitro* maturation

Oocytes were cultured in maturation medium as described by Deb et al. [13]. In brief, COCs were washed three times in maturation medium (TCM-199) supplemented with 10% (v/v) FBS, 1 mg/ml oestradiol-17β, 10 mg/ml follicle-stimulating hormone, 0.6 mM cysteine and 0.2 mM sodium pyruvate, and then transferred to a 4-well plate containing 700 ml of IVM media for 22–24 h at 38.5°C in a humidified atmosphere of 5% CO_2_ in air.

### *In vitro* fertilization and *in vitro* culture

*In vitro* matured COCs were fertilized with frozen-thawed bovine sperm as described by Deb et al. [13]. Semen was thawed at 39°C for 1 min and sperm were washed and pelleted in D-PBS by centrifugation at 750 × ***g*** for 5 min at room temperature. The pellet was diluted with 500 μl of heparin (20 μg/ml) prepared in *in vitro* fertilization (IVF) medium (Tyrodes lactate solution supplemented with 6 mg/ml BSA, 22 mg/ml sodium pyruvate, 100 IU/ml penicillin and 0.1 mg/ml streptomycin) and incubated at 38.5°C in a humidified atmosphere of 5% CO_2_ in air for 15 min (to facilitate capacitation). Thereafter, sperm were diluted in IVF medium (final density of 1×10^6^ sperm/ml). Matured oocytes were transferred to IVF medium (600 μl) containing sperm for 18–20 h. After IVF, cumulus cells were removed by pipetting, and denuded zygotes were placed in 700 μl of CR1-aa medium [14] supplemented with 44 μg/ml sodium pyruvate, 14.6 μg/ml glutamine, 10 μl/ml penicillin–streptomycin, 3 mg/ml BSA and 310 μg/ml glutathione for 3 days. Presumed zygotes were then cultured until Day 8 of embryonic development (Day 0=day of IVF) in medium of the same composition, except that BSA was replaced with 10% (v/v) FBS. Day 8 blastocysts were washed three times in TL-HEPES, transferred to fixative [4% (v/v) paraformaldehyde prepared in 1 M PBS] and stored at 4°C until cells were counted (Figure 3).

### Terminal deoxynucleotidyl transferase dUTP nick-end labelling

Terminal deoxynucleotidyl transferase dUTP nick-end labelling (TUNEL) was performed according to the manufacturer's protocol using an *In Situ* Cell Death Detection Kit (Roche Diagnostics Corp.). Briefly, fixed embryos (*n*=68) were washed twice with 0.3% (w/v) polyvinylpyrrolidone (PVP) prepared in 1 M PBS (PVP–PBS) before being permeabilized [0.5% (v/v) Triton X-100 and 0.1% (w/v) sodium citrate] for 30 min at room temperature [13]. After permeabilization, embryos were washed twice with PVP–PBS and incubated in the dark with fluorescently conjugated terminal deoxynucleotide transferase dUTP for 1 h at 37°C. TUNEL-stained embryos were then washed with PVP–PBS and incubated in PVP–PBS containing 10 μg/ml Hoechst 33342 for 10 min. After washing with PVP–PBS, blastocysts were mounted on to glass slides and their nuclear configuration was analysed. The number of cells per blastocyst was determined by counting Hoechst-stained cells under an epifluorescence microscope (Olympus IX71) equipped with a mercury lamp. TUNEL-positive cells were bright red, indicating the occurrence of apoptosis.

### Immunofluorescence analysis

Bovine embryos were fixed in 4% paraformaldehyde solution. Fixed embryos were washed twice with 0.01 M PBS for 10 min. Embryos were incubated in proteinase K solution at 37°C for 5 min. After washing with PBS, embryos were incubated in blocking solution (D-PBS containing 5% non-immune goat serum and 0.3% Triton X-100) for 1 h. Embryos were incubated overnight with primary antibodies including mouse-derived anti-heat shock protein 70 (HSP70), anti-NF-κB (Santa Cruz Biotechnology) and anti-8-oxoguanosine (8-OxoG; Millipore, Santa Cruz Biotechnology), and then with secondary FITC- and TRITC-conjugated antibodies (diluted 1:50 in D-PBS; Santa Cruz Biotechnology) at room temperature for 90 min. DAPI was used as a counterstain for 5 min. Embryos were mounted on glass slides with Prolong antifade reagent (Molecular Probes). All stained embryos were examined using a confocal laser-scanning microscope (Flouview FV 1000, Olympus).

### mRNA extraction and cDNA reverse transcription

The quantitative RT-PCR was performed according to Deb et al. [13]. In brief, mRNA was extracted from Day 8 blastocysts using a Dynabeads mRNA direct kit (Dynal AS). Embryos were re-suspended in 100 μl lysis buffer and vortexed at room temperature for 2 min. Pre-washed Dynabeads oligo (dT; 20 μl) were mixed with lysate and annealed by rotating for 3 min at room temperature. The Dynal MPC magnetic particle concentrator was used to remove the supernatant. The hybridized mRNA and oligo (dT) magnetic beads were washed twice with 300 μl washing buffer A and twice with 150 μl washing buffer B. To denature and remove secondary structures, bound mRNAs were re-suspended with 8 μl 10 mM Tris/HCl and heated at 65°C for 5 min, followed by rapid quenching on ice for 3 min. The mRNA samples were reverse transcribed into first-stand cDNA using Superscript III first strand reverse transcriptase (Invitrogen). The mRNA samples were transferred into a 200 μl Eppendorf tube containing 1 μl oligo (dT) and 1 μl dNTP mixture (10 mM), incubated at 65°C for 5 min, and then placed on ice for 2 min. The cDNA synthesis mixture (10 μl) and 1 μl Superscript III reverse transcriptase (200 unit/μl) was added to a 200 μl tube containing RNA mixture and incubated at 50°C for 50 min. The reaction was terminated by heating at 85°**C** for 5 min and chilled on ice for 2 min. Subsequently, 1 μl RNase H was added per tube and incubated at 37°**C** for 20 min. The final reaction volume was increased to 80 μl by adding RNase free water.

### Real-time PCR analysis of target genes

**C**andidate genes (HSP70 and NF-κB) were analysed by RT-PCR. Quantitative RT-PCR was performed in duplicate using a CFX98 instrument (Bio-Rad Laboratories) with a 12 litres reaction mixture containing 0.2 mM of each bovine-specific primer (Table 1), 1× iQ SYBR Green Supermix (iQ SYBR Green Supermix kit, Bio-Rad Laboratories) and 1.5 litres diluted cDNA. All cDNA samples were subjected to RT-PCR using glyceraldehyde-3-phosphate dehydrogenase (GAPDH) primers to test for any variation in the expression of this internal control gene. After confirming that there were no significant differences in the relative expression of GAPDH among samples, all transcripts were quantified using independent RT-PCR reactions. The cycling conditions were as follows: 95°C for 3 min, followed by 44 cycles of 15 s at 95°C, 20 s at 57°C and 30 s at 72°C, and a final extension of 5 min at 72°C. Amplification was followed by melting curve analysis using progressive denaturation, during which time the temperature was raised from 65 to 95°C at a transition rate of 0.2°C/s. Continuous fluorescence measurements were acquired during incremental heating. Final quantitative analysis was performed by the ΔΔC(t) method, and results were reported as the relative expression or *n*-fold difference to the calibrator after normalization of the transcript to the average value of the endogenous control, GAPDH. The coefficients of variation (CV) of the intra- and inter-assay variance were calculated according to the formula S.D./mean × 100 for all genes profiled with RT-PCR.

**Table 1:** Forward and reverse primer pairs designed for quantitative RT-PCR

Gene	Primer sequence	Accession no.	Product size (bp)
HSP70	F: GAGTCGTACGCCTTCAACAT	U09861	94
	R: ACTTGTCCAGCACCTTCTTC		
NF-κB	F: TGGCGGAATTACCTTCCATAC	DQ464067	110
	R: CATCACTCTTGCCACAACTTTC		
GAPDH	F: ATTTTGAATGGACAGCCATC	NM173979	120
	R: TGTACAGGAAAGCCCTGACT		

### Statistical analysis

Results are expressed as percentages (%). Transformed data and total cell numbers per blastocyst were analysed using Prism 5. Significant differences between groups were detected using the simple *t* test and one-way ANOVA. *P*<0.05 was considered significant.

## RESULTS

### Cleavage and developmental rates of embryos generated from oocytes treated with various concentrations of coagulansin-A

**Figure 2:**
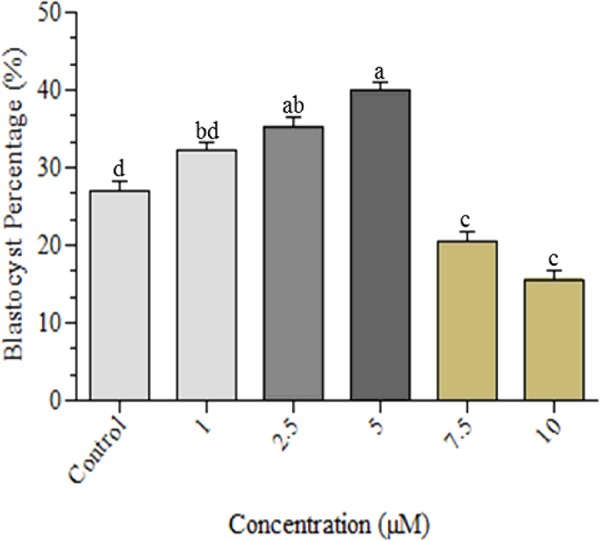
Blastocyst development Columns with different superscripts are significantly different *(P* < 0.05).

The percentage of cleaved embryos was determined on Day 3 of culture. The cleavage rate was greater in the group treated with 5 μM coagulansin-A (90.31%) than in the control group (86.75%), but this was not statistically significant. The percentage of Day 8 blastocysts was greater (*P*<0.05) in the 5 μM coagulansin-A treated group (40.01%) than in the 1, 2.5, 7.5 or 10 μM coagulansin-A groups and the control group (32.56, 35.12, 20.7 15.59 and 27.30% respectively) (Figure 2).

### Determination of the total number of cells and the number of apoptotic cells per blastocyst

**Figure 3:**
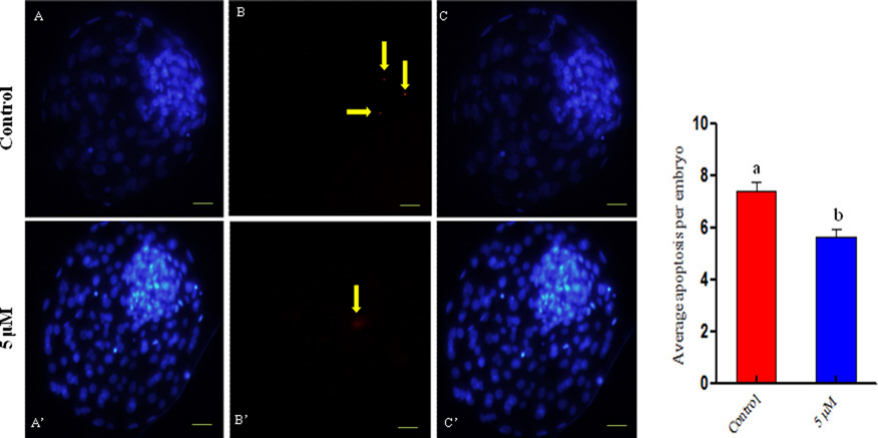
Representative images of bovine embryos stained with Hoechst 33342 Apoptotic cells were identified by TUNEL. The corresponding images were merged. (**A**–**C**) Control group. (**A**′–**C**′) Group treated with 5 μM coagulansin-A. Scale bar, 100 μm. Apoptotic cells are indicated by yellow arrows. The graph shows the number of apoptotic cells per embryo. Columns with different superscripts are significantly different (*P*<0.05).

The total number of cells per Day 8 blastocyst was greater (*P*<0.05) in the 5 μM coagulansin-A group (150.1±3.624) than in the control group (127.7±4.161) (Table 2 and Figure 3). The number of apoptotic cells per Day 8 blastocyst was lower (*P*<0.05) in the 5 μM coagulansin-A group (5.667±0.2873) than in the control group (7.400±0.3754) (Table 2 and Figure 3).

**Table 2:** Characteristics of Day 8 blastocysts in the two groups*

Treatment	No. of blastocysts examined	Total no. of cells per blastocyst	No. of apoptotic cells per blastocyst
Control	15	127.7±4.161^a^	7.400±0.3754^a^
5 μM coagulansin-A	15	150.1±3.624^b^	5.667±0.2873^b^

* Total number of cells and number of apoptotic cells per blastocyst are shown (mean±S.E.M.). ^a,b^Values with different superscripts in the same column are significantly different (*P*<0.05).

### Coagulansin-A-induced activation of HSP70 in bovine embryo development

**Figure 4:**
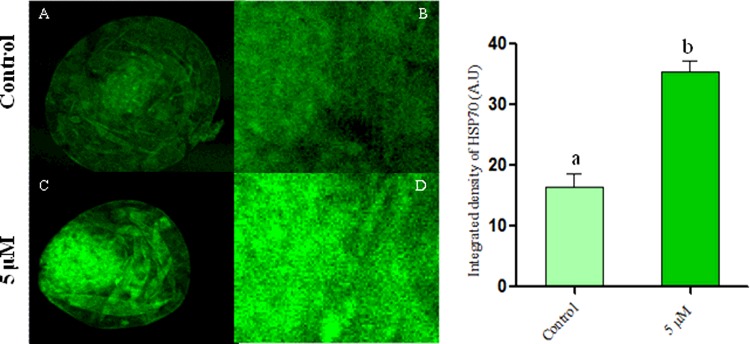
Confocal microscopy, images of bovine embryos *in vitro* (**A** and **B**) Control group. (**C** and **D**) Coagulansin-A treated group. Columns with different superscripts are significantly different *(P* < 0.05).

Among the three concentrations of coagulansin-A tested, only 5 μM had beneficial effects on bovine embryos (Table 3). We performed immunofluorescence to analyse the possible involvement of HSP70 induction in the mechanism by which coagulansin-A affects embryo development. This indicated that integral optical density (IOD) of HSP70 was significantly greater (*P*<0.05) in the 5 μM coagulansin-A group than in control group (Figure 4).

**Table 3:** Cleavage and developmental rates of embryos generated from oocytes treated with various concentrations of coagulansin-A

Coagulansin-A concentration (μM)	Total no. of oocytes	No. of presumed zygotes	No. of cleaved embryos (%)	No. of blastocysts (%)
Control	355	317	275 (86.75)	90 (27.30)^d^
1	355	310	279 (90.00)	102 (32.56)^bd^
2.5	355	316	267 (84.49)	111 (35.12)^ab^
5	355	320	289 (90.31)	120 (40.01)^a^
7.5	355	318	239 (75.15)	66 (20.7)^c^
10	355	314	228 (72.61)	50 (15.59)^c^

^a,b,c,d^Values with different superscripts are significantly different (*P*<0.05).

### Coagulansin-A reduces NF-κB protein expression during bovine embryo development

**Figure 5:**
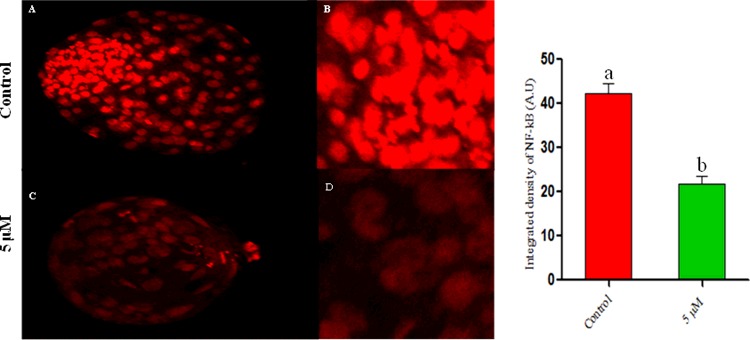
Confocal microscopy (**A** and **B**) Control groups. (**C** and **D**) Coagulansin-A treated groups. Columns with different superscripts are significantly different *(P* < 0.05).

The expression level of NF-κB protein was investigated by performing immunofluorescence analysis. Expression (IOD) of NF-κB protein was greater in control embryos than the coagulansin-A embryos, because treatment with 5 μM coagulansin-A induced HSP70 activation which inhibit NF-κB protein expression during embryo development (Figure 5).

### Coagulansin-A reduces oxidative stress by inhibiting 8-OxoG expression during bovine embryo development

**Figure 6:**
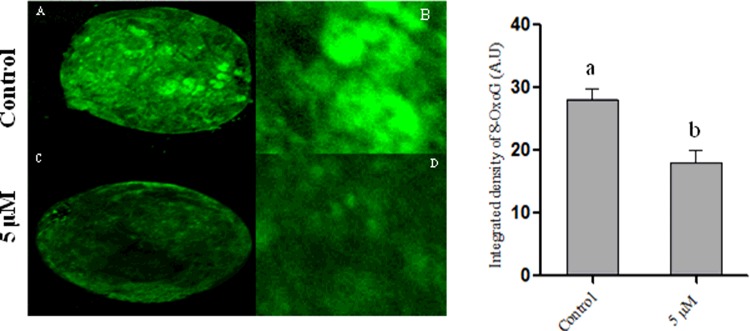
Confocal microscopy of 8-OxoG (**A** and **B**) Control embryos. (**C** and **D**) Coagulansin-A treated embryos. Columns with different superscripts are significantly different (*P*<0.05).

The 8-OxoG is a well-known marker of oxidative stress. To investigate whether coagulansin-A treatment reduces the level of ROS in bovine embryo development, we performed immunostaining of 8-OxoG. Surprisingly, expression (IOD) of 8-OxoG was significantly lower in the 5 μM coagulansin-A group than in control group, which suggests that coagulansin-A inhibit oxidative stress by reducing the expression level of 8-OxoG (Figure 6).

### Gene expression profile in control and coagulansin-A treated groups

**Figure 7:**
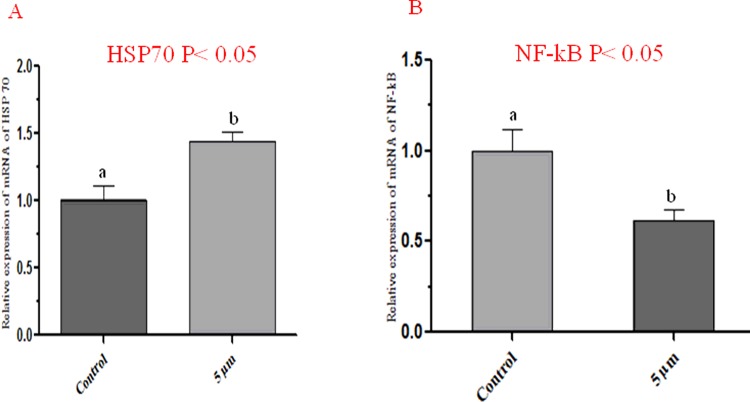
Relative mRNA expression levels in control and coagulansin-A treated blastocysts by RT-PCR.

The expression of two candidate genes HSP70 and NF-κB were also conformed through RT-PCR. Expression amounts were normalized against a housekeeping gene, GAPDH. The expression of HSP70 is greater in coagulansin-A treated group than control group. Similarly the expression of NF-κB was reduced by coagulansin-A than control group (Figure 7).

**Figure 8:**
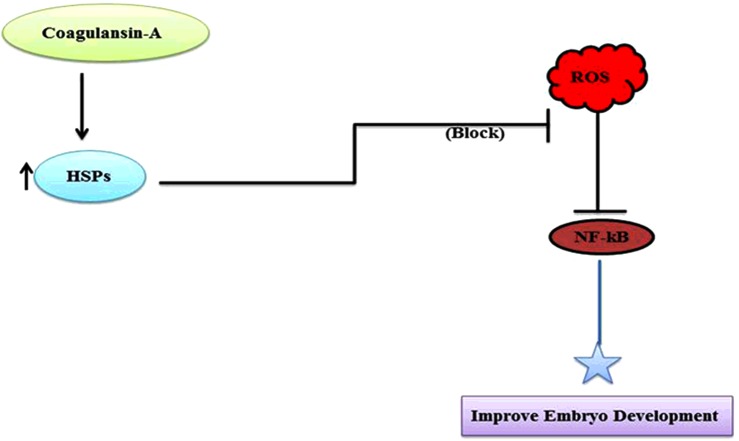
Summary and proposed pathway

## DISCUSSION

The selection of competent oocytes is extremely important for assisted reproductive technologies (ARTs). A number of studies reported that embryonic development depends upon the culture conditions and oocyte quality [15,16]. In this regard one study also reported that the blastocyst development can also be improved by decreasing the O_2_ concentration of the incubator [17]. We reporting for the first time that coagulansin-A supplementation in ART have beneficial effects on embryo development *in vitro*. In the present study we found that among the different concentrations only 5 μM of coagulansin-A treatment more significantly improve the blastocyst development (40.01%) than the control group (27.30%). Consistent with a previous report [18], there was no significant difference in the cleavage rate between the treated group and control group embryos.

Embryo quality is a key factor for successful embryo implantation [19]. Both the cell number and apoptosis play an important role in embryo quality because an increase in apoptosis significantly reduces the total cell number in blastocysts and reduces blastocyst quality [20]. In the present study, the total number of cells per blastocyst was greater in the coagulansin-A treated group than in the control group. Similarly, the number of apoptotic cells per blastocyst was lower in the coagulansin-A treated group than the untreated group (control group). We propose that the improvement in embryo quality is due to coagulansin-A-induced HSP70 activation, which improved the blastocyst quality.

Heat shock proteins act as cellular antioxidants [21]. Most cells produce these proteins, which belong to the HSP70 family [22]. HSP70 protects cells from adverse effects in stress conditions [23,24] and functions as a molecular chaperone in the absence of stress [25,26]. HSP70 also worked as a defensive protein against ROS during the inflammation [27]. The HSP70 level increases as bovine embryos develop *in vitro* [28]. In the present study we found that the coagulansin-A induced the HSP70 expression which is in agreement with a previous report [7], and this protein after inducing in the culture system improves the embryo quality and efficiency.

The NF-κB pathway plays an important role in many developmental and cellular processes [29]. NF-κB is activated at specific stages of murine spermatogenesis and induces the transcription of various genes in testes [30]. NF-κB activation is also extremely important for the development of mouse embryos beyond the 2-cell stage [31]. In murine and bovine oocytes, changes in NFκBIA and IKBa occur, which are related to aging [32,33]. Similarly, NF-κB pathway activation is also involved in various inflammatory diseases [34]. In the present study treatment of coagulansin-A in IVM medium reduces the activation of NF-κB pathway in bovine embryo, which is in agreement with a previous report [35].

For embryo development, the balance between ROS and scavengers (antioxidants) is extremely important [36]. ROS causes the dysfunction of DNA, RNA and proteins [37], and affect sperm and oocyte fusion [38]. The ROS also causes the induction of inflammation as it activates and induces NF-κB pathway [39]. The 8-OxoG is produced as a result of ROS and is commonly used to determine the ROS concentration [40]. Currently we found that the expression of 8-OxoG was greater in control embryos than coagulansin-A treated embryos, indicative of increased ROS concentration in control than treated group.

In previous literature it is already reported that culture condition effect the expression level of various genes of blastocyst [41,42]. In the present study supplementation of the IVM medium with coagulansin-A increases the expression of HSP70 which is in agreement with [7]. NF-κB transcription factor is activated by ROS [39]. In the present study, we also found that the HSP70 induction by coagulansin-A reduces the ROS which ultimately down-regulate the expression of NF-κB transcription factor which is in agreement with [39].

We conclude that addition of coagulansin-A, a steroidal lactone, to IVM media induces HSP70 expression in bovine embryos, which reduces the ROS concentration, inhibits NF-κB activation and improves embryo development. When NF-κB is down-regulated, inflammatory markers such as inducible nitric oxide synthase, COX-2 and Toll-like receptor 4 are also down-regulated which needs further confirmation.

## Abbreviations

ART: assisted reproductive technology
COC: cumulus–oocyte complex
COX: cyclooxygenase
GAPDH: glyceraldehyde-3-phosphate dehydrogenase
HSP70: heat shock protein 70
IOD: integral optical density
IVC: *in vitro* culture
IVF: *in vitro* fertilization
IVM: *in vitro* maturation
NF-κB: nuclear factor-κB
8-OxoG: 8-oxoguanosine
PVP: polyvinylpyrrolidone
ROS: reactive oxygen species
RT-PCR: real-time PCR
TUNEL: terminal deoxynucleotidyl transferase dUTP nick-end labelling
